# Adaptation of an in-vitro digestion model with different zinc-supplementation strategies on nutrient degradation of piglets

**DOI:** 10.1016/j.heliyon.2024.e33300

**Published:** 2024-06-19

**Authors:** Annegret Lucke, Annette Liesegang, Dolf Kümmerlen, Michael Czarniecki, Brigitta Annette Wichert

**Affiliations:** a Institute of Animal Nutrition and Dietetics, Vetsuisse Faculty, University of Zurich, 8057, Zurich, Switzerland; b Division of Swine Medicine, Department of Farm Animals, Vetsuisse Faculty, University of Zurich, 8057, Zurich, Switzerland; c Solid Chemicals GmbH, Gossau, Switzerland

**Keywords:** In-vitro digestion, Piglet, Zinc, In-situ bags, Daisy

## Abstract

In-vitro studies are widely used in nutrition research. Two major challenges using in-vitro models in animal nutrition research are the individual adaptation of in-vitro digestion models to varying physiological conditions and small digesta output limiting sample material for further analysis. Since several years, the use of zinc in animal production has been legally reduced to control zinc emissions. Earlier, zinc doses around 3000 mg/kg diet were used to prevent post-weaning diarrhea and promote growth in weaning piglets. The first aim of this study was to adapt an in-vitro digestion system for piglets with increased sample output. The second aim was to study the effect of a titanium-bound zinc source at legal dietary inclusion levels on nutrient degradation in an in-vitro digestion model. The experiment was conducted in a 2x2 factorial design incubating 2 different feeds (1. control feed: a commercial piglet diet containing 75 mg zinc per kg diet and 2. treatment feed: control feed with 50 mg of a titanium-bound zinc oxide) in in-situ digestion bags in the Ankom Daisy® incubator with or without digestive enzymes (pepsin, pancreatic enzymes and bile salts). Residuals of incubated feed were analyzed for crude ash, crude protein and starch. The addition of pepsin, pancreatic enzymes and bile salts significantly increased organic matter, crude protein and starch degradation from the digested feed, therefore making the distinction of nutrient disappearance due to enzyme activity versus due to dissolution possible. In conclusion we established an in-vitro digestion model to evaluate the effect of addition of a new zinc source on the enzymatic digestion in piglets. However, addition of the new zinc source did not significantly improve nutrient degradation in the in-vitro digestion model.

## 1 Introduction

In 2003, the European Food Safety Authority (EFSA) proposed a reduction of dietary zinc (Zn) levels in complete feed to reduce Zn emissions originating from animal production into the environment [1]. Up to that date, doses between 2500 and 3000 mg/kg diet were commonly used in piglets to avoid post-weaning diarrhea [2]. The newly proposed maximum Zn levels for complete diets were 200 mg/kg complete feed for dogs and cats, 180 mg Zn/kg diet for salmonids and milk replacers for calves, 150 mg Zn/kg complete feed for piglets, sows, rabbits and all fish other than salmonids and 120 mg Zn/kg complete feed other species and categories, which are currently in use in the European Union [3]. These limits currently are the same for all authorized Zn compounds. The trace element zinc plays a vital role in most cell communication and metabolic pathways, is present in >300 zinc-dependent enzymes and is a part of many transcription factors [4]. Research in rats has shown that dietary zinc supply affects digestive enzyme activity [5].

A new Zn compound based on Zn oxide bound to a titanium oxide nucleus (TRT) to increase surface was used in this study [6,7]. The zinc compound further comprises a hydrophobic lipid coating, protecting the zinc oxide from reactions in the stomach. This zinc compound is expected to improve growth and feed conversion rates in piglets achieving similar results as previously used high zinc oxide doses [6,7]. Previous internal research of the company developing TRT had shown that the titanium-bound zinc increases the digestive properties of lactase and pepsin (unpublished data). The suspected mechanism is a local difference of the pH on the surface of titanium bound zinc molecules which increase the lactase activity in-vitro (unpublished data). However, this increase in lactase activity was only studied at neutral pH and therefore has to be verified under physiological conditions in-vitro. The pepsin activity has been tested at pH = 3 in glycin buffer (unpublished data). The knowledge gap was whether the observed increases in enzyme activities could be translated into improved nutrient digestion parameters in a more physiological setting such as an in-vitro digestion model mimicking both the gastric and the intestinal digestion step, too.

In-vitro digestion systems are widely used to study the digestibility of macronutrients such as proteins, as well as bioavailability of micronutrients such as trace elements and phytochemicals [8]. The major advantages of in-vitro digestion systems compared to animal experiments include their cost-effectiveness, user-friendliness, potential for standardization and therefore high reproducibility. Moreover, in-vitro digestion systems are a valuable tool to overcome potential ethical concerns related to in-vivo experiments [9,10]. In human nutrition, efforts were made to harmonize the great variety of static in-vitro digestion methods [11,12] and the resulting protocol was validated with an in-vivo pig digestion model [13]. While established in-vitro digestion models for microbial ruminal such as the Rusitec typically use sample volumes large enough to complete a full proximate analysis of the digesta of numerous replicates [14,15], the sample volume after in-vitro digestion in in-vitro monogastric digestion models mimicking gastric and small intestinal digestion, are typically much lower. Earlier research showed that a simple pepsin-pancreatin digestion procedure can be used to in-vitro digest feed samples in commercially available in-situ digestion bags in larger in-vitro batch incubation systems to increase sample throughput to study protein degradation [16]. Especially during the weaning period, the gastrointestinal tract of piglets undergoes some physiological and metabolic changes, specifically showing higher stomach pH values [17]. These changes may on the one hand interfere with the resistance of the piglet to infection with pathogens. On the other hand, they impair nutrient digestibility and therefore should be accounted for in in-vitro digestion models.

Therefore, the aim of this study was the adaptation of a static in-vitro system using in-situ digestion bags to increase sample output. In this system, we specifically aimed to simulate exclusively endogenous processes of gastric and small intestinal digestion in piglets post-weaning. In addition, we aimed to study the degradation of organic matter (OM), in particular protein and starch, depending on the use of a new titanium bound zinc-source. Our hypothesis was that the zinc source can positively impact nutrient digestion in piglets post-weaning. Moreover, we hypothesized that the in-vitro digestion model allows for differentiation of nutrient degradation due to enzymatic digestion versus loss due to solubility.

## 2 Materials and methods

### 2.1 Experimental design and experimental diets

Two piglet feeds were used as a substrate in this in-vitro digestion trial. The control (CON) diet was supplemented with 75 mg zinc (15 mg from zinc oxide and 60 mg from glycine-zinc chelate hydrate) per kg diet. The background zinc level from the dietary ingredients of the diets was 65 mg zinc per kg diet. The CON diet was a commercial piglet feed (Ferkel maxi Plus, 8–25 kg) obtained from Granovit AG (Kaiseraugust, Switzerland). The treatment (TRT) diet consisted of the CON diet supplemented with 50 mg of an additional zinc source with a titanium dioxide nucleus per kg diet [6,7]. The labelled analytical composition of both diets included 15.8 % crude protein (CP), 4.8 % crude fiber, 5 % crude fat (CF) and 5.1 % crude ash (CA). The dietary composition of both diets was identical with exception of different zinc sources and included the following ingredients in descending order: Wheat, barley, wheat bran, toasted soya beans, soybean meal, brown rice, dried apple pulp, beef and pork fat, sugar beet molasses, sugar beet pulp, locust bean meal, sodium chloride, dicalcium phosphate, monocalcium phosphate, calcium carbonate, fructo-oligosaccharides, corn. Both diets were supplemented with the following feed additives (amount per kg feed) 12000 IE vitamin A, 1800 IE vitamin D3, 80 mg copper; 15 mg zinc (zinc oxide), 60 mg zinc (glycine-zinc chelate hydrate), 48 mg manganese, 0.3 mg selenium, 120 mg iron, 1 mg iodine, 2.1 g methionine, 150 U Endo-1,3(49-beta-Glucanase, 1220 U Endo-1,4-beta-xylanase, 2000 FTU 6-phytase, 2e9KBE *Saccharomyces cerevisiae*, 146 mg Saccharinsodium.

Both feeds were tested either with or without addition of gastric, pancreatic enzymes and bile salts in a static in-vitro digestion system to simulate endogenous processes of gastric and small intestinal digestion. The experiment was conducted as a randomized 2x2 experimental design with diet (CON, TRT) and digestion enzyme supplementation (buffer without digestion enzymes (−) or buffer with digestion enzymes (+) as experimental factors resulting in the following four treatments: CON+, CON-, TRT+, TRT-.

### 2.2 Feed sample preparation and measurement of enzyme activities

The feed was ground in a Cyclotec 1093 mill (Foss GmbH, Hamburg, Germany) to pass a 1 mm screen. Before conducting the in-vitro digestion experiment, enzyme activities were determined in pepsin and pancreatin. Pepsin activity was determined as suggested by Brodkorb et al. based on a spectrophotometric method adapted from Anson et al. [18,19]. Lipase activity was measured by a pH titration method as detailed by Brodkorb et al. [12] based on [[20], [21], [22], [23], [24]]. Trypsin activity was determined with a kinetic spectrophotometric rate determination method as described by Brodkorb et al. [12], modified from Hummel [25].

### 2.3 In-vitro digestion procedure

We adapted the Infogest static in-vitro simulation of the gastric and small intestinal digestion [12] for the Daisy incubator system (Ankom, Fairport, NY). Two experimental runs with 4 incubation bottles each were conducted, representing the four different treatments. In each digestion bottle, 30 nylon bags (50 μm pore size, R510, Ankom, Fairport, NY) filled with 5 g of diet [16] were incubated simultaneously at 39 °C to increase sample output. An in-vitro digestion procedure to simulate consecutive gastric and small intestinal digestion was performed.

The gastric digestion buffer was prepared according to Brodkorb et al. [12] and contained 6.9 mM KCl, 0.9 mM KH_2_PO_4_, 25 mM NaHCO_3_, 47.2 mM NaCl, 0.12 mM MgCl_2_(H_2_O)_6_, 0.5 mM (NH_4_)_2_CO_3_, and 0.15 mM CaCl_2_(H_2_O)_2._ The pH was adjusted to 2.4 using 6 M HCl. The pH after the gastric digestion phase was in the range of 5.1–5.3. Therefore the in-vitro incubation covered the gastric pH range of piglets at the timepoint of weaning to two weeks after weaning [17,26,27]. In the CON+ and TRT + groups, pepsin (P7012, Sigma-Aldrich) was added at a concentration of 2000 U/ml to the gastric digestion buffer. Each daisy incubation bottle contained 2 L of digestion fluid. Feed samples were incubated for 2 h at 39 °C in the gastric digestion buffer with or without pepsin, pancreatic enzyme and bile salt supplementation. Afterwards the gastric digestion buffer in the Daisy incubation bottles was completely exchanged to the intestinal digestion buffer.

The small intestinal digestion buffer contained 6.8 mM KCl, 0.8 mM KH_2_PO_4_, 85 mM NaHCO_3_, 38.4 mM NaCl, 0.33 mM MgCl_2_(H_2_O)_6_, and 0.6 mM CaCl_2_(H_2_O)_2_ and was adjusted to pH = 7.1 [12]. In the CON+ and TRT + groups, porcine pancreatin (P7545, Sigma-Aldrich) and porcine bile (B8631, Sigma-Aldrich) were added at a concentration of 100 U trypsin per ml final digestion buffer and 10 mM for bile salts to the small digestion buffer [12]. The resulting concentrations for lipase was 21293 U lipase per ml final digestion buffer. Feed samples were incubated for 2.5 h at 39 °C in the small intestinal digestion buffer. Incubation times were based on transit times observed in in-vivo experiments [28,29]. After the digestion procedure, the nylon bags were immediately dried in a hot-air oven at 103 °C overnight to stop further digestive enzyme activity.

### 2.4 Feed and digesta analysis

Feed samples and in-vitro digesta samples were analyzed for DM by oven-drying at 103 °C overnight. DM was recorded for each incubated nylon bag (N = 240, n = 60 per treatment). The content of 5 nylon bags were pooled for further analyses to achieve sufficient sample material for further analyses (N = 48, n = 12 per treatment). The CA content was analyzed by ashing samples in duplicate in a muffle oven at 550 °C overnight. The CP content was analyzed in duplicate according to the official methods [30]. Total starch content was measured with a commercial enzymatic assay kit K-TSTA (Megazyme International Ireland Ltd., Bray, Ireland) [31]. OM was calculated as OM (% DM) = 100 – CA (% DM). Nutrient degradation was calculated as follows: [(nutrient in feed (g per pool) – nutrient in residual (g per pool))/nutrient in feed (g per pool)] x 100 %.

### 2.5 Statistical analysis

All statistical analyses were conducted using the R statistical programming language (version 4.3.1) in R studio (version 2023.12.0.369) [32,33]. Data were analyzed with a linear mixed-effects model using the lme4-package version 1.1.35.1 [34]. The model included feed type, enzyme supplementation and their interaction as fixed effects and experimental run and incubation bottle as random effects. Posthoc tests were conducted using the Tukey adjustment in the lsmeans package version 2.30.0 [35]. The Shapiro-Wilk test was used to test for normal distribution of the residuals. Summary statistics including, mean, median, standard error and confidence intervals were calculated using the dplyr package version 1.1.3 [36] and the broom package, version 0.2.9.4 [37]. Figures were created using the ggplot2 package version 3.4.4 [38]. A p-value of p < 0.05 was considered significant and 0.05 ≤ p < 0.10 as a tendency.

## 3 Results

### 3.1 Effect of enzyme supplementation on nutrient degradation

The analytical composition of in-vitro digested feed is shown in Table 1.

**Table 1:** Analytical composition of feed before in-vitro digestion.

	CON	TRT
CP (% DM)	16.5	16.9
Starch (% DM)	35.7	34.1
CA (% DM)	5.7	5.9

CON: piglet feed supplemented with 75 mg zinc (15 mg from zinc oxide and 60 mg from glycine-zinc chelate hydrate) per kg diet, TRT: CON feed supplemented with 50 mg zinc oxide with a titanium nucleus per kg diet. CA: crude ash, CP: crude protein, DM: dry matter.

**Fig. 1:**
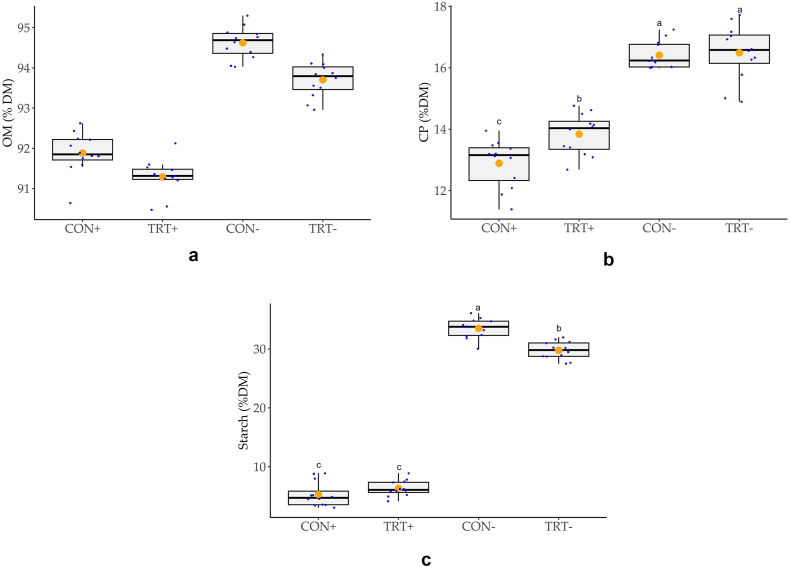
a–c. Effects of feed type and enzyme supplementation on (**a**) organic matter (OM) content in dry matter (%) of in-vitro digesta, (**b**) crude protein (CP) content in dry matter (%) of in-vitro digesta, and (**c**) starch content in dry matter (%) of in-vitro digesta, respectively (n = 12). Orange dots ● represent the mean of the data. Superscripts a-c indicate different pairwise comparisons in case of a significant interaction effect of feed type x enzymes (p < 0.001). CON: piglet feed supplemented with 75 mg zinc (15 mg from zinc oxide and 60 mg from glycine-zinc chelate hydrate) per kg diet, TRT: CON feed supplemented with 50 mg zinc oxide with a titanium dioxide nucleus per kg diet; -: incubation without digestive enzyme supplementation, +: in-vitro incubation with gastric, pancreatic enzymes and bile salts.

OM, CP and starch content in in-vitro digested feed (% DM) are displayed in Fig. 1a–c. Enzyme supplementation decreased the OM portion in digesta from 94.2 % to 92.6 % OM in DM (p < 0.001, Fig. 1a). Moreover, addition of enzymes decreased CP content in in-vitro digested feed significantly (p < 0.001) from 16.5 % CP in DM in groups without digestive enzyme supplementation to 13.4 % CP in DM in the groups with digestive enzyme supplementation (Fig. 1b). The starch content in feed residuals was reduced from 31.6 % to 5.8 % in DM by enzyme supplementation (p < 0.001, Fig. 1c).

**Fig. 2:**
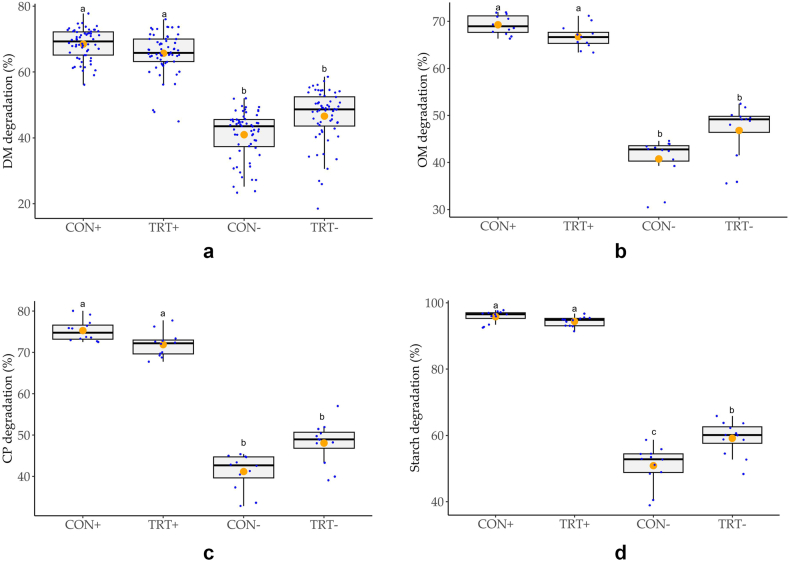
a–d. Effects of feed type and enzyme supplementation on (**a**) in-vitro dry matter (DM) degradation (n = 60), (**b**) in-vitro organic matter (OM) degradation, (**c**) in-vitro crude protein (CP) degradation, and (**d**) in-vitro starch degradation (n = 12). Orange dots ● represent the mean of the data. Superscripts a-b indicate significantly different pairwise comparisons in case of a significant interaction effect of feed type x enzymes (p < 0.001). CON: piglet feed supplemented with 75 mg zinc (15 mg from zinc oxide and 60 mg from glycine-zinc chelate hydrate) per kg diet, TRT: CON feed supplemented with zinc oxide with a titanium dioxide nucleus; -: incubation without digestive enzyme supplementation, +: in-vitro incubation with gastric, pancreatic enzymes and bile salts.

DM, OM, CP and starch degradation of in-vitro incubated feed are displayed in Fig. 2a–d, respectively. Enzyme addition to the digestion mix increased DM degradation from 43.8 % to 67.1 % and increased OM degradation from the digestion bags from 43.8 % to 68.0 % (p < 0.001, Fig. 2a). The results indicate a difference of 23.3 % or 24.2 % in DM and OM degradation respectively, which can be attributed to the digestive enzyme addition (Fig. 2b). CP degradation increased by 29.2 % from 44.7 % to 73.9 % when digestion enzymes were added to the incubation buffers (Fig. 2c). Starch degradation increased by 40.1 % from 55.0 % to 95.1 % by enzyme addition (p < 0.001, Fig. 2d).

### 3.2 Effect of zinc source and interaction of zinc source and enzyme supplementation on nutrient degradation

OM in DM in the residuals of in-vitro incubated feed was significantly higher in CON group (93.2 % OM) compared to 92.5 % OM in DM in TRT group (p = 0.001), irrespective of enzyme addition (Fig. 1a). In enzymatically incubated samples, no difference between CON and TRT in OM in DM was found. Concurrently, OM enzymatic degradation was not affected by the feed type (p > 0.05, Fig. 1a).

Overall, CP in DM in the residuals of in-vitro incubated feed was significantly lower in CON compared to TRT (p = 0.001) and specifically was lower in the CON group compared to the TRT group (p = 0.049), when the feed was enzymatically digested (Fig. 1b). Concurrently, the CP degradation was numerically lower in the enzymatically digested TRT feed compared to the CON feed (p = 0.443, Fig. 2c).

Overall starch in DM of in-vitro incubated feed was not affected by the type of feed. However, a significant interaction term suggested that starch in feed residuals of the CON group without enzymatic incubation was higher than in the TRT group without enzyme incubation (Fig. 1c), which was reflected by a lower starch degradation in the CON group without enzymatic incubation (Fig. 2d). In the enzymatically digested feed, starch degradation did not differ between the two diets.

## 4 Discussion

In the present study we developed an adapted in-vitro digestion model to study the effect of different feedstuffs in piglets post-weaning. By comparing enzyme supplemented digestion fluids with gastric and intestinal buffers without enzyme supplementation, we were able to determine CP, starch, OM and DM degradation which could be directly related to the digestion enzyme activity as opposed to nutrient disappearance due to dissolution. Moreover, the batch incubation system allowed us to increase sample throughput. This enabled us to test the effect of a new titanium-bound zinc source on the nutrient degradation. The different zinc sources did not affect nutrient degradation in the digestion bags in enzymatically digested feed. Therefore, the hypothesis that the new titanium-bound zinc oxide source improves enzymatic digestion was not supported by our data.

To allow the collection of sufficient digesta material for subsequent analyses, we adapted the Infogest static digestion model [12] by incubating the feed material in in-situ bags in the Daisy batch incubation system [16]. The Daisy incubation system was chosen in our study, as it allows for larger sample throughput. Previous research has shown that up to 30 nylon bags filled with each 5 g of feed sample can be used in this system to estimate protein digestibility without affecting the results [16,39]. The static in-vitro digestion model that served as a basis for the Infogest method was initially validated focusing on protein digestion with an in-vivo pig trial using pigs with an average body weight of 50 kg, therefore older than weaning age [[11], [12], [13]]. In-vivo digestibility data in weaning piglets are scarce. However, data of in-vitro protein degradation in our model with in-vivo ileal digestibility data in pigs (40–60 kg) are comparable to the study of Boisen et al. who found 70 %, 75 % and 78 % protein digestibility in barley, wheat and soybean meal, which were the main ingredients of our diet [40]. Data in weaning piglets show, that the standardised ileal protein digestibility is significantly decreased in the first week of weaning and gradually rebuilds until week 3 post-weaning, irrespective of protein source. The average ileal digestibilty of protein in weaning piglets fed a diet with wheat as protein source was 74 %, which is close to our observed result [41]. Starch degradation observed in our in-vitro study was very close to 95.8 % ileal digestibility of starch reported for weaning piglets 10 days post-weaning [42]. Dry matter degradation in our study was in the range of values (64 %, measured at the fourth quarter of the small intestine) reported in piglets 3 weeks after weaning at the age of 3 weeks [43]. Overall, the present in-vitro model successfully showed the selective decrease of OM and CP in the digestion bags. Enzyme concentrations in this study were chosen to match the established Infogest method after measuring the enzyme activity in pancreatin, bile extract and pepsin [12] to ensure repeatability and all enzymatic components of this in-vitro model originate from pigs. Earlier studies in piglets have shown that enzyme activity might be affected by the weaning process, for example observing decreased lipase and trypsin activities and increased amylase activity at weaning with 21 days of age [44]. Another study observed temporal decreases of trypsin, chymotrypsin, amylase and lipase activities in the week after weaning with 28 d of age [45]. Care has to be taken in interpretation of enzyme activity results across studies as they usually are expressed as activity per weight of pancreas and moreover activities were evaluated with a variety of methods [[44], [45], [46]].

In our study the OM and CP degradation due to digestive enzyme activity increased by 24 and 29 %, respectively. This does not imply that nutrient degradation due to diffusion from the nylon bag are not actually digested in-vivo but allows to study the treatment effects on enzymatic digestion and the quantification of these diffusion losses.

In comparison to other in-vitro as well as in-vivo methods such as in-situ or mobile nylon bag technique models, our model allows the estimation of nutrient degradation which is purely due to enzymatic degradation, while excluding potential effects of intestinal microbiota and possible effects on nutrient uptake by the host [47].

Sams et al. (2016) emphasize the relevance of pH and lipase for in-vitro models of gastric digestion, noting that most models use very low pH values that are not representative of the fed conditions [48]. Indeed, we adapted our model to meet the higher gastric pH values after weaning as compared to adult pigs and the original Infogest in-vitro digestion method, which increased steadily due to the buffering capacity of the ingested food [17]. Ideally, the gastric digestion step would include the use of gastric lipase, which however has only a minor contribution due to a 6000-times less activity compared to pancreatic lipase in lipid digestion in piglets [[49], [50], [51]]. Moreover, at the time the experiments were performed, only gastric lipase from rabbits was available on the market and its use would have increased the cost of the in-vitro essays considerably [12]. As previously mentioned research is not fully clear about its role in the porcine digestion process, we therefore decided not to include this enzyme in our experiment [49]. Nevertheless, in case of future availability of porcine gastric lipase the inclusion into the experimental set up is recommended to closer mimic the in-vivo situation.

We did not observe differences in nutrient degradation between the enzymatically digested feeds. The effect of adding the titanium-bound zinc source on nutrient digestion was evaluated in a more complex environment including 1) the presence of a complete diet for piglets and 2) the incubation at physiological relevant pH-, buffer- and temperature conditions. Under these conditions the previously in water at pH 7 or glycine buffer at pH 3 measured increases of lactase or pepsin activities (unpublished material) may not be detectable. A previous study in rats has shown that feeding a high zinc supply diet (234 mg Zn/kg diet) improves the activity of maltase, pepsin, pancreatic amylase and protease [5]. However, it has to be mentioned that this equals approximately 5-times the zinc concentration as mentioned for the zinc adequate diet and therefore should be interpreted cautiously [5]. Moreover, the previously detected effects of the titanium-bound zinc on pig performance [6,7] may also be related to other mechanisms such as improvement of mucosa-associated enzyme activities such as maltase and isomaltase and potential effects on the gastrointestinal microbiota and intestinal barrier [52]. Furthermore, a potential effect of TRT on enzymatic digestion in-vitro might only become evident if the enzyme shortage is a limiting factor in the digestion process. Therefore, an area of future research might include testing TRT at different enzyme concentrations in the existing in-vitro model.

Limitations of this in-vitro digestion model compared to in-vivo experiments in piglets in include that the uptake across the intestinal wall and subsequent metabolism of the titanium-bound zinc compound cannot be studied. On the other hand, the model allows to specifically study the effect on enzymatic digestion. It would however be important to determine the bioavailability of the newly developed zinc compound in piglets [53]. Moreover, digesting the feed in-vivo might improve the access of digestion enzymes to the feed compared to the use of the digestion bags in-vitro and provide different mechanic stimuli during the digestion process. Nevertheless, the use of digestion bags in-vitro has been well established previously and the amount of feed used in the bags in our study was previously shown to be adequate in a comparable system [16]. At the moment, it is to be determined how much of the titanium-bound zinc portion is absorbed in-vivo. For this reason, we chose an experimental design where the titanium-bound zinc source is added to the CON diet which itself already contained a zinc supplementation. The amount of titanium-bound zinc source was specifically chosen to ensure comparability with previously conducted internal research [6,7]. Moreover, using the same commercial diet batch as a basis for CON and TRT diet allowed us to avoid additional variations in the feed composition. Due to the relatively high content of native zinc in the diets, the TRT diet exceeded the currently applicable maximum level of zinc in piglets in the EU [3]. However, despite the relatively high zinc content in the TRT diet, based on the present results, it cannot be concluded that the titanium-bound zinc source exerts positive effects on nutrient digestion in piglets. Therefore, future experiments should include studying the effect of the titanium-bound zinc compound on mucosal enzyme activities, intestinal barrier and gastrointestinal microbiota. Furthermore, future research should aim to study the effect of different dosages of the titanium-bound zinc source in comparison to alternative zinc supplementation strategies in-vitro and in-vivo.

## 5 Conclusions

The present study describes the adaptation of an in-vitro piglet digestion model allowing to indirectly study the digestion products of enzymatically digested food while excluding microbial or absorptive components of digestion. The model was used to indirectly study the potential effect of different zinc sources on digestive enzyme activity of gastric and pancreatic origin by measuring in-vitro nutrient degradation. Adding a titanium-bound zinc source to the commercial piglet feed did not affect in-vitro digestion parameters significantly. However, a possible influence on small-intestinal mucosal enzymes and microbes in the hindgut should be investigated in the future.

## Ethical statement

Not applicable.

## Funding

This work was funded by 10.13039/501100013348Innosuisse (Swiss Innovation Agency Life Sciences, Einsteinstrasse 2, 3003 Bern, Switzerland) Innovationsprojekt, grant number 42943.1-IL-LS.

## Data availability statement

Data will be made available on request.

## CRediT authorship contribution statement

**Annegret Lucke:** Writing – review & editing, Writing – original draft, Visualization, Validation, Software, Methodology, Investigation, Formal analysis, Data curation, Conceptualization. **Annette Liesegang:** Writing – review & editing, Writing – original draft, Supervision, Software, Resources, Methodology, Funding acquisition, Conceptualization. **Dolf Kümmerlen:** Writing – review & editing, Resources, Project administration, Funding acquisition. **Michael Czarniecki:** Writing – review & editing, Resources, Funding acquisition. **Brigitta Annette Wichert:** Writing – review & editing, Writing – original draft, Supervision, Resources, Project administration, Methodology, Funding acquisition, Conceptualization.

## Declaration of competing interest

The authors declare the following financial interests/personal relationships which may be considered as potential competing interests: Brigitta Wichert reports financial support was provided by 10.13039/501100013348Innosuisse Swiss Innovation Agency. Dolf Kuemmerlen reports financial support was provided by 10.13039/501100013348Innosuisse Swiss Innovation Agency. Michael Czarniecki reports a relationship with Solid Chemicals GmbH that includes: employment and funding grants. If there are other authors, they declare that they have no known competing financial interests or personal relationships that could have appeared to influence the work reported in this paper.
